# Temporal instability and differences in injury severity between restrained and unrestrained drivers in speeding-related crashes

**DOI:** 10.1038/s41598-023-36906-7

**Published:** 2023-06-16

**Authors:** Chamroeun Se, Thanapong Champahom, Panuwat Wisutwattanasak, Sajjakaj Jomnonkwao, Vatanavongs Ratanavaraha

**Affiliations:** 1 Institute of Research and Development, Suranaree University of Technology, 111, University Avenue, Suranaree, Muang Nakhon Ratchasima, 30000 Thailand; 2 Department of Management, Faculty of Business Administration, Rajamangala University of Technology Isan, 744 Sura Narai Rd, Nai-Muang, Muang Nakhon Ratchasima, 30000 Thailand; 3 School of Transportation Engineering, Institute of Engineering, Suranaree University of Technology, 111, University Avenue, Suranaree, Muang Nakhon Ratchasima, 30000 Thailand

**Keywords:** Civil engineering, Risk factors

## Abstract

Upon detecting a crash impact, the vehicle restraint system locks the driver in place. However, external factors such as speeding, crash mechanisms, roadway attributes, vehicle type, and the surrounding environment typically contribute to the driver being jostled within the vehicle. As a result, it is crucial to model unrestrained and restrained drivers separately to reveal the true impact of the restraint system and other factors on driver injury severities. This paper aims to explore the differences in factors affecting injury severity for seatbelt-restrained and unrestrained drivers involved in speeding-related crashes while accounting for temporal instability in the investigation. Utilizing crash data from Thailand between 2012 and 2017, mixed logit models with heterogeneity in means and variances were employed to account for multi-layered unobserved heterogeneity. For restrained drivers, the risk of fatal or severe crashes was positively associated with factors such as male drivers, alcohol influence, flush/barrier median roadways, sloped roadways, vans, running off the roadway without roadside guardrails, and nighttime on unlit or lit roads. For unrestrained drivers, the likelihood of fatal or severe injuries increased in crashes involving older drivers, alcohol influence, raised or depressed median roadways, four-lane roadways, passenger cars, running off the roadway without roadside guardrails, and crashes occurring in rainy conditions. The out-of-sample prediction simulation results are particularly significant, as they show the maximum safety benefits achievable solely by using a vehicle's seatbelt system. Likelihood ratio test and predictive comparison findings highlight the considerable combined impact of temporal instability and the non-transferability of restrained and unrestrained driver injury severities across the periods studied. This finding also demonstrates a potential reduction in severe and fatal injury rates by simply replicating restrained driver conditions. The findings should be of value to policymakers, decision-makers, and highway engineers when developing potential countermeasures to improve driver safety and reduce the frequency of severe and fatal speeding-related single-vehicle crashes.

## Introduction

Death and serious injuries due to roadway crashes remain a weighty concern for low and middle-income developing countries, where nine out of the ten dead victims are found. Compared to developing countries from the rest of the globe, Thailand was among the top ten countries with the highest fatalities rate due to road accidents, with an annual average death rate of 32 per 100,000 population between 2011 and 2016 and approximately 56 deaths per day or over 20,000 people being killed in road accidents each years^1^. According to the World Bank report, in 2016, the estimated cost of death and serious injuries due to road accidents in Thailand was $44.71 billion which is equivalent to 10.9% of the country’s Gross Domestic Product (GDP)^2^. While speeding violation is the main cause of the majority of crashes in Thailand, a high rate of unrestrained drivers (seatbelt) among drivers (42%) and front passengers (60%) remain the national public health problems^1^.

According to the statistics from Thailand’s Department of Highway, single-vehicle run-off roadway crashes not only account for the highest frequency rate but also the highest number of fatalities, compared to other crash types such as rear-end, sideswipe, and head-on crash^3^. Between 2012 to 2017, approximately 77% of these single-vehicle crashes were caused by drivers exceeding the speed limit and were responsible for about 76% of the death and serious injuries^4^. While unsafe speed is the cause of the majority of roadway crashes on road due to greater loss of vehicle control risk, the majority of these drivers may be intrinsically unsafe drivers. That is, evidently, 61.8% of drivers involved in single-vehicle speeding-related crashes (between 2012 and 2017 in Thailand) were not restrained using a seatbelt when the crashes occurred. These drivers are the ones who are prone to death or serious injury in crashes due to their unsafe driving habits^5^. Due to such significant impacts, investigations of crashes involving speeding and seatbelt violation are of considerable importance.

Speeding can cause numerous safety issues namely, a reduction in the effectiveness of occupant protection equipment (seatbelts, airbags, and crumple zones) and road safety structures (road friction, guardrail, and median divider), and an increase in stopping distance after the driver perceives danger and crash severity^6^. The seatbelt is one part of the vehicle restraint systems (aside from airbag and crumple zones) that takes part in absorbing kinetic energy in collisions, thereby reducing the force involved and subsequently reducing the risk of death or serious injury in a crash. Upon sensing the impact generated by the collision, the vehicle’s seatbelt system is triggered by locking the driver in place, preventing the driver from tumbling and hitting the objects in or outside of the vehicle, whereas other external factors such as speeding, crash mechanism, roadway attributes (e.g., curve or slope alignments), vehicle type, and surrounding environment all contribute to moving driver out of the driver’s seat and tumbling driver around inside or even outside the vehicle. Additionally, a combination of unrestrained driver and speeding behavior is found to be strongly associated with an increase in the probability of higher injury severities level in single-vehicle crashes^7^. These facts suggest that single-vehicle crashes involving seatbelt restrained-driver and unrestrained-driver should be separately examined to uncover the true effect of the seatbelt restraint system and other associated risk factors on driver-injury severities, particularly speeding-related crashes.

## Literature review

### Review of previous single-vehicle crash-injury severity studies

Table 1 provides a review of previous research publications on single-vehicle crash-injury severity since 2010. A total of 52 studies were found and reviewed. As seen in Table 1, some earlier studies investigated the contributing factors to injury severity in single-vehicle crashes using aggregate crash data^7–15^. In contrast, other research studies analyzed single-vehicle crash-injury severities using disaggregated data; for example, crashes on divided/undivided urban road^16^, crashes on rural/urban roadways^17^, crashes involving unimpaired/alcohol-impaired/drug-impaired drivers^18^, crashes with one-/two-/three-occupants^19^, riders/drivers of the crashes^20^, crashes on 2-lane/4-lane roadway^21^, crashes with difference light/weather condition^22^, familiar/unfamiliar drivers of the crashes^23^, passenger car/SUV crashes^24^, crashes under different weather scenarios^25, 26^, fixed-object/overturn crashes^27^, crashes on arterial/ secondary/branch roadway ^28^, and crashes from different period (temporal instability)^4, 29–34^. However, none of the aforementioned literature investigated single-vehicle crashes using disaggregated data concerning restrained and unrestrained drivers, while also accounting for their speeding violation behavior in the crashes.

**Table 1:** A review of methodologies, research target/sub target and problems addressed for previous studies on single-vehicle crash-injury severity analysis since 2010.

Paper	Type of data aggregation/disaggregation	Methodologies	Heterogeneity/transferability/temporal instability/predictive comparison
Jung et al.^41^	Single-vehicle crashes in **rainy weather**	Polychotomous response model	(None)
Rifaat et al.^42^	**Urban neighborhood** single-vehicle crashes	Logistic regression model	(None)
Chen and Chen ^43^	**Truck drivers** of single-/multi-vehicle crash	Mixed logit model	Heterogeneity
Xie et al.^44^	**Rural** single-vehicle crashes	Latent class logit and Mixed logit model	Heterogeneity
Bham et al.^16^	Single-vehicle crashes on **divided/undivided urban** **highways**	Generalized logistic regression model	Transferability
Jiang et al.^8^	Overall single-vehicle crashes	Zero-inflated ordered probit models	(None)
Kim et al.^9^	Overall single-vehicle crashes	Mixed logit model with heterogeneity in means	Heterogeneity
Dissanayake and Roy ^10^	Overall single-vehicle crashes	Binary logit model	(None)
Xiong et al.^11^	Overall single-vehicle crashes	A Markov switching approach with road-segment heterogeneity	Heterogeneity
Weiss et al.^45^	**Young drivers** of single-/two-vehicle crashes	Mixed logit model	Heterogeneity
Wang and Qin ^46^	Overall single-vehicle crashes	Structural Equation Modeling	(None)
Wu et al.^47^	Drivers of single-/multi-vehicle crashes on **rural two-lane highways**	Mixed logit model	Heterogeneity, Transferability
Behnood and Mannering^29^	**Yearly data from 2004–2012** of single-vehicle crashes	Mixed logit model	Heterogeneity, Transferability, Temporal instability
Lee and Li^48^	Drivers of single-/two-vehicle crashes	Boosted regression trees	(None)
Naik et al.^49^	**Truck-involved** single-vehicle crashes	Mixed ordered model and Mixed logit model	Heterogeneity
Wu et al.^17^	**Rural/Urban roadways** single-vehicle crashes	Nested logit model and Mixed logit model	Heterogeneity, Transferability
Anarkooli et al.^12^	Overall single-vehicle crashes	Random-effects generalized ordered probit model	Heterogeneity
Behnood and Mannering^18^	**Unimpaired/Alcohol-impaired/Drug-impaired drivers** of single-vehicle crashes	Mixed logit model	Heterogeneity, Transferability
Behnood and Mannering^19^	**One-occupant/two-occupants/three-occupants** of single-vehicle crashes	Mixed logit model with heterogeneity in means	Heterogeneity, Transferability
Li et al.^50^	**Low-visibility** single-vehicle crashes	Finite mixture random parameters model	Heterogeneity
Osman et al.^51^	**Commercially-licensed drivers** of single-vehicle crashes	Mixed generalized ordered response probit model	Heterogeneity
Li et al.^13^	Overall single-vehicle crashes	Finite mixture random parameters model	Heterogeneity
Hou et al.^14^	Overall single-vehicle crashes	Mixed logit model with heterogeneity in means	Heterogeneity
Yu et al.^15^	Overall single-vehicle crashes	Latent class random parameters model	Heterogeneity, Temporal instability
Dabbour et al.^30^	**Yearly data from 2007–2013** of single-vehicle crashes	Mixed ordered model	Heterogeneity, Temporal instability
Zhou and Chin^20^	**Riders/drivers** of single-vehicle crashes	Ordered probit model	Transferability
Se et al.^21^	**2-lanes/4-lanes roadway** single-vehicle crashes	Multinomial logit model	Transferability
Fountas et al.^22^	**Daylight/darkness**, **Lighted/unlighted roadways**, **Fine/poor weather**, of single-vehicle crashes	Zero-inflated hierarchical ordered probit approach with correlated disturbances	Heterogeneity, Transferability
Wen and Xue^23^	**Familiar/unfamiliar drivers** in single-vehicle crashes **on mountainous highways**	Random-effects generalized ordered probit models	Heterogeneity, Transferability
Rahimi et al.^36^	**Truck-involved** single-vehicle crashes	Random thresholds random parameters hierarchical ordered probit model	Heterogeneity
Khan and Vachal^7^	Overall single-vehicle crashes	Generalized ordered logit model	(None)
Chen et al.^52^	**Rural highway** single-vehicle crashes	Latent class binary logistic regression model	Heterogeneity
Se et al.^53^	Overall single-vehicle crashes	Multinomial logit model	(None)
Wen et al.^24^	**Passenger car/SUV rollover** single-vehicle crashes	Mixed ordered logit model	Heterogeneity, Transferability
Yu et al.^25^	**Yearly data from 2010–2016** of Single-vehicle crashes under **Clean/overcast/raining/fog/crosswind/blowing sand weather types**	Mixed logit model	Heterogeneity, Temporal instability
Yu et al.^31^	**2014/2015/2016–2017** single-vehicle crashes	Random thresholds random parameters hierarchical ordered probit	Heterogeneity, Transferability, Temporal instability
Yu et al.^54^	**Yearly data from 2010–2016** of single-vehicle crashes	Fusion convolutional neural network with random term	Heterogeneity, Temporal instability
Se et al.^32^	**Yearly data from 2011–2017** of single-vehicle crashes	Correlated random parameters model with heterogeneity in means and variances	Heterogeneity, Transferability, Temporal instability
Roque et al.^27^	**Fixed object/overturn** single-vehicle crashes	Mixed logit model	Heterogeneity, Transferability
Yan et al.^55^	Overall single-vehicle crashes	Tree-based models and non-Tree-based models	(None)
Hosseinpour and Haleem ^56^	**Large truck-involved** single-vehicle crashes	Mixed ordered probit model	Heterogeneity
Wei et al.^26^	**Foggy weather/clear weather** single-vehicle crashes	Mixed logit model	Heterogeneity, Transferability
Se et al.^4^	**2012–2013/2014–2015/2016–2017** single-vehicle crashes involving **non-speeding/speeding-related crashes**	Random parameters model with heterogeneity in means and variances	Heterogeneity, Transferability, Temporal instability
Yan et al.^33^	**Rural** single-vehicle crashes involving **alcohol-impaired driving**, and **yearly data from 2014–2018**	Random parameters model with heterogeneity in means and variances	Heterogeneity, Transferability, Temporal instability
Islam et al.^39^	**Truck-involved** single-vehicle crashes on **curved segments/ straight segments**	Random parameters model with heterogeneity in means and variances	Heterogeneity, Transferability
Yu and Long^37^	**Rollover** single-vehicle crashes	Random thresholds random parameters hierarchical ordered logit model	Heterogeneity
Yan et al.^38^	Overturned and hit-fixed-object crash on **rural road by speeding driving**, and **yearly data from 2015 to 2018**	Correlated random parameters model with heterogeneity in means	Heterogeneity, Transferability, Temporal instability Predictive comparison
Alnawmasi and Mannering ^40^	Single-/two-vehicle crash, and **yearly data from 2009–2013 separately**	Random parameters model with heterogeneity in means and variances	Heterogeneity, Transferability, Temporal instability Predictive comparison
Ma et al.^57^	Overall single-/multi-vehicle crashes	Partial proportional odds model	Heterogeneity, Transferability
Yan et al.^34^	**Nighttime** single-vehicle crashes considering **young drivers/ Middle-age drivers/ Old drivers**, and yearly data from 2014–2017 separately	Random parameters model with heterogeneity in means and variances	Heterogeneity, Transferability, Temporal instability Predictive comparison
Cai and Wei ^58^	Overall single-vehicle crashes	Bayesian random parameters multinomial model	Heterogeneity
Cai and Wu ^28^	**Arterial/Secondary/Branch roadway** single-vehicle crashes	Spatiotemporal interaction logit (STI-logit) model	Heterogeneity, Transferability

In terms of the methods employed in the previous studies listed in Table 1, it is evident that a broad array of methodological approaches has been adopted in research over the past decade. Nevertheless, the use of random parameters models (such as mixed logit or ordered models) has been the most popular, likely because of their flexibility in capturing the heterogeneous effects of risk factors across crash populations. This flexibility leads to improved prediction accuracy, better model fit, and more reliable conclusions^35^. Additionally, Table 1 also showed that there are multiple variants of the random parameters model used for the single-vehicle crash-injury severity in the recent years, including random parameters model that allows for possible heterogeneity in means^9, 14, 19^, random thresholds random parameters hierarchical ordered probit model^36, 37^, correlated random parameters with heterogeneity in means^32, 38^, and random parameters model that allows both heterogeneity in means and variances^4, 32–34, 39, 40^.

### Review of the effect of speeding and seatbelt on the injury severity

While numerous empirical studies have explored the impact of speeding (as an explanatory variable) on crash injury severities^9, 20, 42, 59–62^, only a few have examined the effect of speeding violations at a disaggregated level. Renski et al.^63^ investigated the influence of speed limit increases on crash injury severity in single-vehicle crashes, using indicators for road segments with speed limit changes from 88.5 to 96.6 kph, 88.5 to 104.6 kph, and 104.6 to 112.7 kph as part of the explanatory variables in their statistical model. On the other hand, Alnawmasi and Mannering ^40^ examined the consequences of higher speed limits on the frequency and severity of freeway crashes, using data from before and after speed limit increases separately. They discovered that the factors affecting driver-injury severities had changed before and after the speed limit increase in one- and two-vehicle crashes. In another study, the temporal instability of contributing factors between speeding-related and non-speeding-related crashes was investigated, revealing significant differences between the influencing factors of both models^4^. Although using excessive speeding as explanatory variable, Abegaz et al.^61^ identified varying coefficients for speeding's impact on different injury levels, with the most significant effects on severe and fatal crashes. Focusing on speeding-related rural crashes, Yan et al.^38^ studied rural overturned and hit-fixed-object crashes and found temporal shifts and non-transferability between these two types of crashes.

On the other hand, the effect vehicle’s seatbelt restraint system on the crash injury severity was also extensively explored in the previous studies and found positive safety effect of seatbelt on the outcome severity of the crashes while using it as an explanatory variable^4, 17, 44, 64^. Only a few studies have hypothesized and proved that the effect of seatbelt use status may not be exogeneous, but may be endogenous to crash-related injury severity^5, 65^. Interestingly, Eluru and Bhat ^5^ found that safety-conscious drivers are more likely to wear seat belts, and their defensive habits also lead to less severe injuries when they are involved in crashes, whereas, Abay et al.^65^’s finding revealed that belted drivers offset the safety benefits that accrue from using a seat belt by driving more aggressively. In another study, Shimamura et al.^66^ focuses on the tendency of front seat occupants to sustain severer injuries due to forward movement of passengers in rear seats at the moment of car-to-car frontal collisions, and evaluates the effectiveness of rear passengers’ wearing seat belts in reducing injuries of front seat occupants. They found that the number of killed or seriously injured passengers in front seats was estimated to decrease by 28% if unbelted rear seat occupants come to wear seat belts. Additionally, only one study by Abu-Zidan et al.^67^ examined the effects of seatbelt usage on injury patterns and outcomes for restrained vehicle occupants compared to unrestrained occupants using Chi-square tests. Their results indicated that injury scores for the thorax, back, and lower extremity were significantly higher in unrestrained patients than in restrained patients.

### Research gap and contributions of the current study

Table 1 also shows that some recent single-vehicle crash-injury severity studies have investigated heterogeneity, transferability, and temporal instability, as well as undertaken predictive comparisons^34, 38, 40^. However, none of them have focused on speeding-related crashes among seatbelt-restrained and unrestrained drivers, respectively. Therefore, based on the thorough reviews mentioned above, the current study is among the first of its kind to identify the unobserved heterogeneity, transferability, and temporal instability of contributing factors (including driver characteristics, roadway attributes, vehicle types, crash characteristics, and environmental characteristics) related to speeding-related single-vehicle crashes among seatbelt-restrained and unrestrained drivers, respectively. In addition, this study is the first to conduct a predictive comparison between within-sample and out-of-sample predictions to observe the aggregate effects of temporal shifts in speeding-related crashes for both types of drivers, as well as the aggregate differences in injury severity probability among restrained and unrestrained drivers.

In this regard, the current study's concept is novel as it offers four distinct contributions: (1) disaggregating the overall single-vehicle speeding-related crashes by seatbelt restraint system use status and providing an explicit understanding of whether the determinants of speeding-related crashes are transferable across restrained and unrestrained drivers; (2) examining the temporal instability of speeding-related crashes for restrained and unrestrained drivers, respectively; (3) investigating the differences and potential heterogeneity in multiple determinants affecting speeding-related crashes across restrained and unrestrained drivers; (4) disentangling the predictive differences resulting from temporal instability and seatbelt use status differences. By incorporating these aspects, this paper enhances the understanding of the role of seatbelt usage in the progression of speeding-related crash injury severities over time and provides valuable insights for practitioners and policymakers in developing targeted interventions and strategies to reduce speeding-related crash severities for drivers with diverse seatbelt usage behaviors.

### Empirical setting

This research study was conducted using the highway run-off-road single-vehicle crash dataset extracted from the Highway Accident Information Management System, Department of Highway (DOH), Thailand. The present study focused on and analyzed single-vehicle crashes related to speeding. As per the DOH, a crash is classified as speeding-related if the police officer determines that the driver's violation of the speed limit was the primary cause of the crashes. Similarly, according to Liu and Chen^68^ and NHTSA^69^, crashes are considered speeding-related when the involved driver is cited for an offense related to speeding, engaging in a race, driving at an inappropriate speed for the prevailing conditions, or surpassing the posted speed limit. The timeline of the crash dataset was from January 1st, 2012 to December 31st, 2017. There was a total of 6837 speeding-related single-vehicle crash cases. 4223 or 61.8% of the drivers were unrestrained with a seatbelt when the crash happened, whereas only 2614 or 38.2% of the drivers were restrained. In terms of injury severity distribution, unrestrained drivers resulted in 14% fatalities and 14.4% severe injuries. As expected, restrained drivers resulted in only 10.8% fatalities and 14 severe injuries.

In the original crash data from DOH ^70^, the police office employed a three-level injury severity scale for all crash records, which included minor injury (covering both minor injuries and property damage only (PDO) crashes), severe injury (involving drivers hospitalized for over three weeks), and fatal injury (drivers killed at the crash scene or at the hospital). Consequently, this study also considered three levels of driver injury severities: minor injury, severe injury, and fatal injury. The explanatory variables extracted from the original dataset can be classified into five categories: driver characteristics (gender, driver age, and DUI (drivers under influence of alcohol)), road characteristics (median type, number of lanes, work zone, pavement types, road alignment, intersection, and U-turn), vehicle types (van, passenger car, pick-up truck, and large truck), crash characteristics (run-of-road with/without hitting the guardrail, mounting traffic island), and environmental and temporal characteristic (nighttime, unlit road, lit road, weekend, morning peak-hour, and evening peak-hour). The presence of multicollinearity within the dataset was assessed by examining the Pearson correlation coefficients for the independent variables. Most pairs had a correlation value below 0.7, suggesting that multicollinearity was not an issue^71, 72^. However, the pairs [two-lane, four-lane], [curve; RORCG], [passenger car; pick-up car], and [wet road; raining] displayed correlations greater than 0.7. Consequently, the indicators for two-lane, curved roads, pick-up cars, and wet roads were removed to address the multicollinearity concerns.

In this research, three distinct timeframes are identified, specifically 2012–2013, 2014–2015, and 2016–2017. This categorization was derived from the outcomes of the temporal instability test (refer to the subsequent section), which indicates that the biennial groupings exhibit strong temporal fluctuations. This classification approach not only guarantees that volatility is not overlooked due to the aggregation of time but also helps prevent the problem of inadequate data that may arise from shorter durations^73^. Based on the examination of the temporal features, the entire dataset is divided into six subsets: restrained drivers from 2012 to 2013, unrestrained drivers from 2012 to 2013, restrained drivers from 2014 to 2015, unrestrained drivers from 2014 to 2015, restrained drivers from 2016 to 2017, and unrestrained drivers from 2016 to 2017. Table 2 presents the descriptive statistics and frequencies for all explanatory variables utilized in the analysis

**Table 2:** Descriptive statistics of the significant explanatory variables.

Variables		2012–2013		2014–2015		2016–2017	
		Restrained	Unrestrained	Restrained	Unrestrained	Restrained	Unrestrained
**Dependent Variable: Injury severities**		Count(%)	Count(%)	Count(%)	Count(%)	Count(%)	Count(%)
Minor injury		583(75.42)	887(72.23)	759(75.98)	1141(70.96)	622(73.87)	996(71.81)
Severe injury		108(13.97)	181(14.74)	143(14.31)	231(14.37)	115(13.66)	197(14.20)
Fatal injury		82(10.61)	160(13.03)	97(9.71)	236(14.68)	105(12.47)	194(13.99)
**Independent variables**	Mean(SD)	Mean(SD)	Mean(SD)	Mean(SD)	Mean(SD)	Mean(SD)	
Young	1 = Age less than 26 years old; 0 = Otherwise	0.095(0.294)	0.096(0.294)	0.239(0.426)	0.271(0.445)	0.193(0.395)	0.203(0.402)
Old	1 = Age more than 49 years old; 0 = Otherwise	0.165(0.371)	0.153(0.360)	0.185(0.388)	0.178(0.383)	0.175(0.380)	0.193(0.395)
Male	1 = Male driver; 0 = Otherwise	0.857(0.349)	0.879(0.325)	0.882(0.321)	0.860(0.347)	0.880(0.325)	0.888(0.314)
Alcohol	1 = Driver under the influence of alcohol; 0 = Otherwise	0.015(0.123)	0.005(0.075)	0.013(0.113)	0.006(0.082)	0.011(0.108)	0.005(0.070)
Flush median	1 = Crash on a flush-median road; 0 = Otherwise	0.023(0.150)	0.046(0.210)	0.047(0.211)	0.060(0.238)	0.043(0.205)	0.056(0.230)
Raised median	1 = Crash on a raised-median road; 0 = Otherwise	0.269(0.443)	0.285(0.451)	0.259(0.438)	0.253(0.435)	0.245(0.430)	0.245(0.430)
Depressed median	1 = Crash on a depressed-median road; 0 = Otherwise	0.406(0.491)	0.328(0.470)	0.346(0.476)	0.335(0.472)	0.350(0.477)	0.369(0.482)
Barrier median	1 = Crash on a barrier-median road; 0 = Otherwise	0.029(0.170)	0.027(0.164)	0.047(0.211)	0.044(0.205)	0.080(0.272)	0.040(0.196)
Four-lane	1 = Crash on a four-lane road; 0 = Otherwise	0.645(0.478)	0.570(0.495)	0.648(0.477)	0.588(0.492)	0.654(0.475)	0.587(0.492)
Work zone	1 = Crash on work zone area; 0 = Otherwise	0.025(0.158)	0.019(0.138)	0.019(0.136)	0.019(0.139)	0.027(0.163)	0.021(0.145)
Asphalt	1 = Crash on asphalt pavement; 0 = Otherwise	0.941(0.234)	0.904(0.293)	0.946(0.224)	0.912(0.282)	0.939(0.238)	0.918(0.273)
Curve	1 = Crash on curve road; 0 = Otherwise	0.302(0.459)	0.289(0.453)	0.359(0.480)	0.285(0.451)	0.334(0.472)	0.298(0.457)
Slope	1 = Crash on sloping road; 0 = Otherwise	0.107(0.309)	0.107(0.309)	0.126(0.332)	0.086(0.281)	0.118(0.323)	0.090(0.287)
Intersection	1 = Crash at intersection area; 0 = Otherwise	0.065(0.248)	0.066(0.249)	0.073(0.260)	0.067(0.251)	0.055(0.229)	0.062(0.241)
U-turn	1 = Crash at U-turn area; 0 = Otherwise	0.107(0.309)	0.101(0.302)	0.089(0.285)	0.071(0.257)	0.083(0.276)	0.079(0.270)
Van	1 = Van; 0 = Otherwise	0.019(0.138)	0.017(0.129)	0.017(0.129)	0.020(0.141)	0.024(0.156)	0.020(0.140)
Passenger car	1 = Passenger car; 0 = Otherwise	0.376(0.484)	0.352(0.477)	0.376(0.484)	0.356(0.479)	0.378(0.485)	0.354(0.478)
Truck	1 = Vehicle is large truck; 0 = Otherwise	0.112(0.316)	0.131(0.338)	0.129(0.335)	0.120(0.325)	0.111(0.315)	0.136(0.343)
RORS	1 = Vehicle runs off-road on straight; 0 = Otherwise	0.111(0.314)	0.110(0.313)	0.106(0.308)	0.130(0.337)	0.142(0.349)	0.115(0.319)
RORSG	1 = Vehicle runs off-road on straight and hit guardrail; 0 = Otherwise	0.341(0.474)	0.315(0.464)	0.323(0.467)	0.318(0.466)	0.334(0.472)	0.333(0.471)
RORC	1 = Vehicle runs off-road on a curve; 0 = Otherwise	0.056(0.231)	0.063(0.243)	0.070(0.255)	0.065(0.248)	0.059(0.236)	0.054(0.227)
RORCG	1 = Vehicle runs off the road on a curve and hit guardrail; 0 = Otherwise	0.206(0.405)	0.168(0.374)	0.253(0.435)	0.190(0.393)	0.247(0.431)	0.206(0.404)
MTI	1 = Vehicle mounts traffic island; 0 = Otherwise	0.249(0.433)	0.298(0.457)	0.209(0.406)	0.242(0.428)	0.186(0.389)	0.257(0.437)
Raining	1 = Raining; O = Otherwise	0.249(0.433)	0.223(0.417)	0.239(0.426)	0.228(0.419)	0.289(0.453)	0.231(0.421)
Unlit road	1 = Crash at night on an unlit road; 0 = Otherwise	0.130(0.337)	0.139(0.346)	0.105(0.306)	0.115(0.319)	0.100(0.301)	0.082(0.275)
Lit road	1 = Crash at night on a lit road; 0 = Otherwise	0.368(0.482)	0.330(0.470)	0.368(0.482)	0.365(0.481)	0.342(0.474)	0.390(0.487)
Weekend	1 = Weekend; 0 = Otherwise	0.284(0.451)	0.302(0.459)	0.333(0.471)	0.297(0.457)	0.307(0.461)	0.307(0.461)
Morning peak hour	1 = Morning peak hour (7:00–9:30); 0 = Otherwise	0.085(0.279)	0.060(0.238)	0.066(0.248)	0.080(0.272)	0.072(0.259)	0.089(0.285)
Evening peak hour	1 = Evening peak hour (16:00–19:30); 0 = Otherwise	0.107(0.309)	0.103(0.304)	0.152(0.359)	0.121(0.326)	0.141(0.348)	0.117(0.322)

## Methodology

### Unobserved heterogeneity and crash severity study

Mannering et al.^35^ have provided plausible evidence for why the effect of the considered risk factors may vary across the observation. That is, data collected for the analysis can never be complete. For example, in terms of human characteristic attributes, the crash data may differentiate the gender differences of the victims from each crash, while there are several pieces of unknown information to the analyst that may have great variation across victims with the same gender such as height, weight and reaction times or risk-taking behaviors of the same gender with different ages. Therefore, the assumption that all the male victims, compared to females, are more likely to sustain a particular injury severity (an assumption that all male observations have a fixed effect on injury outcomes probability) could create biased results or incomplete conclusions. An evident example can be seen in the finding of the previous studies. Xin et al.^74^ found that 53.4% of the male occupant were less likely to sustain serious injury, whereas only 46.6% of the male victims were having a higher risk of severe injuries. Similarly, Se et al.^75^ also discovered that 57.1% of male drivers had a higher risk of being killed in the crashes, whereas 42.9% of them were more likely to sustain a minor or severe injury in the crash. While male victims may have higher injury tolerance^76^, a significant cohort of them may also have risk-seeking driving or riding behaviors than female^77^, illustrating a great variance of physiological characteristics and safety and risk awareness among male victims of the crashes^74, 75^.

Not only human elements, but attributes related to vehicle, roadway, traffic, environment, and temporal characteristics also have great variability across the crashes^35^. As can be seen in the Table 1, to account for unobserved heterogeneity, the application of the random parameter (mixed) model and it’s multiple variants, particularly the model extension that allow for means and variances heterogeneity has been adopted in numerous recent insightful articles such as Alogaili and Mannering^78^, Wang et al.^79^, Yan et al.^38^, Alnawmasi and Mannering^40^, Se et al.^80^, Yan et al.^34^, Wang et al.^81^, Islam et al.^82^, Behnood and Mannering^83^, and Hou et al.^84^. The random parameters model with heterogeneity in means and variance was found to be superior than standard random parameters model in crash-injury severity analysis due to its’ the great flexibility in capturing a greater extent of underlying unobserved characteristics, more precise predictions, and better model fit^80, 84–87^. Considering three levels of driver-injury severity outcomes—minor injury, severe injury and fatal injury—this study extensively considered the mixed logit model with heterogeneity in means and variances.

### Model development framework

As indicated earlier, the mixed logit model with heterogeneity in means and variances is used in this study. Theoretically, we need to first define a severity function ${Y}_{in}$ which determines the probability that crash ${\varvec{i}}$ will result in injury-severity level $n$ as follow^88^:

1 $${Y}_{in}={\alpha }_{n}+{{\varvec{\beta}}}_{{\varvec{i}}}{{\varvec{X}}}_{{\varvec{i}}{\varvec{n}}}+{\varepsilon }_{in}$$

where ${\alpha }_{n}$ is a constant specific to injury-severity $n$ (with one of them set to zero for identification), ${{\varvec{\beta}}}_{{\varvec{i}}}$ is a vector of regression coefficients, ${{\varvec{X}}}_{{\varvec{i}}{\varvec{n}}}$ is a vector of exogenous attributes (such as driver-, roadway-, vehicle-, crash-, and environmental characteristics) specific to crash ${\varvec{i}}$ and injury-severity level $n$, and ${\varepsilon }_{in}$ is an error term.

To overcome the strict limitation of the multinomial logit model, the mixed logit relaxes the assumption of the logit model by allowing the parameter coefficients to vary across observations by introducing a mixing distribution. In this study, all the parameters (one at a time) were tested by allowing them to vary across crash observations. If the standard deviation of the tested parameter is not statistically significant (i.e., variance or scale parameter is zero)^75, 89^, then the parameter was not random and the factor would set back to have the same effect across all observation. If the standard deviation is statistically significant, then parameter was a random parameter and its’ parameter coefficient also significantly varied across observation. If none of parameters produce significant standard deviation, the model is fall back to be a standard multinomial logit model. Additionally, the injury-severity probability function of the mixed logit model is defined as follows^88^:

2 $${P}_{i}\left(n\right)=\int \frac{{e}^{{\alpha }_{n+}\left({{\varvec{\beta}}}_{i} {{\varvec{X}}}_{in}\right)}}{\sum_{m}{e}^{{\alpha }_{m+}\left({{\varvec{\beta}}}_{i} {{\varvec{X}}}_{im}\right)}}f\left({\varvec{\beta}}|\rho \right)d{\varvec{\beta}},$$

where all other parameters are previously defined, ${P}_{i}\left(n\right)$ denotes a probability of driver sustaining injury severity *n* in crash $i$, and $f\left({\varvec{\beta}}|\rho \right)$ is a density function of ${\varvec{\beta}}$ with $\rho$ being the vector of parameters of the density function (mean and variance). Various analyst-specified distribution types were used including standard normal, triangular, standard uniform, and lognormal. The final model was selected by comparing the model fit of each utilized distribution.

In the mixed logit model, a variable is referred to as a fixed parameter if the parameter does not vary across observation. If it does vary across observations, it will be regarded as a random parameter (i.e., having a significant standard deviation). Additionally, Seraneeprakarn et al.^86^ suggested that specific crash-level and/or segment-level attributes might affect the mean of a parameter that differs across observations, commonly known as random parameters. Moreover, examining how heterogeneity impacts the variance of a random-parameter distribution, which ultimately establishes parameter values for individual observations, could be a significant consideration. By allowing the variance in the parameter distribution to further delineate the dispersion of parameter values across observations, this method offers more flexibility in capturing the hidden unobserved heterogeneity, potentially enabling greater sensitivity to crash conditions^35, 86^. Consequently, this approach may yield deeper understanding of controllable factors for crash injury-reduction strategies. On the other hand, employing a mere simple distribution to describe the random parameter mean and variance, as is usually done in random parameters models, might not adequately represent the inherent unobserved heterogeneity. In this regard, the model can be more flexible in uncovering the unobserved heterogeneity by allowing the interaction effect between non-random parameters with the mean and variance of the random parameters on the injury-severity probability. Following the previous studies^32, 80, 83, 84, 86^, let ${{\varvec{\beta}}}_{in}$ be a vector of estimable parameters that vary across crash observations, which is derived as:

3 $${{\varvec{\beta}}}_{in}={{\varvec{\beta}}}_{{\varvec{n}}}+{\boldsymbol{\Theta }}_{in}{{\varvec{M}}}_{in}+{\sigma }_{in}EXP({{\varvec{\omega}}}_{in}{{\varvec{S}}{\varvec{D}}}_{in}){\nu }_{in}$$

where ${{\varvec{\beta}}}_{n}$ is a mean parameter estimateed across all crashes^90, 91^, ${{\varvec{M}}}_{in}$ denotes a vector of the variables that capture heterogeneity in the mean that influences injury severity *n,* with parameter vector ${\boldsymbol{\Theta }}_{in}$^86^, ${{\varvec{S}}{\varvec{D}}}_{in}$ is a vector of variables that captures heterogeneity in the standard deviation ${\sigma }_{in}$ with the corresponding vector ${{\varvec{\omega}}}_{in}$^86^, and ${\nu }_{in}$ is a disturbance term.

The structure presented in Eq. (3) enables two distinct attribute vectors (${{\varvec{M}}}_{in}$ and ${{\varvec{S}}{\varvec{D}}}_{in}$) to influence the parameter values that vary across observations (i.e., random parameter)^86^. The vectors ${{\varvec{M}}}_{in}$ and ${{\varvec{S}}{\varvec{D}}}_{in}$ may encompass attributes related to driver, roadway, vehicle, crash, environmental characteristics, or other potential heterogeneity sources. If no variables prove significant ${{\varvec{S}}{\varvec{D}}}_{in}$, the model is a heterogeneity in means only model. Meanwhile, any unobserved heterogeneity not depicted in the form of ${{\varvec{M}}}_{in}$ and ${{\varvec{S}}{\varvec{D}}}_{in}$ results in a mixed logit model without heterogeneity in either means or variances. In this paper, the model estimations were analyzed using a simulated maximum likelihood approach and 1000 Halton draws were found to produce sufficient integration of parameter accuracy and stability.

### Model interpretation

As in recent crash-injury severity studies^38, 78, 80, 92^, the marginal effect is commonly used to interpret the effect of the explanatory variable on the outcome injury severity of the crashes. Theoretically, the marginal effect is the changes in outcome (injury severity) probabilities due to one specific explanatory variable changing the value from 0 to 1 (for binary explanatory variable), while holding other variables unchanged. The average marginal effect over sample observation can be computed as^84, 93^:

4 $$M{E}_{{X}_{i}}^{{P}_{i}(n)}=\frac{1}{m}\sum_{j=1}^{m}[{P}_{i}(n)|\left({X}_{ij}=1\right)-{P}_{i}\left(n\right)|\left({X}_{ij}=0\right)]$$

where $M{E}_{{X}_{i}}^{{P}_{i}(n)}$ is the average marginal effect of the explanatory variable ${X}_{i}$ and ${X}_{ij}$ denotes any specific explanatory variable of the observation *j*.

In this study, the Econometric Software NLOGIT Version 6.0 was utilized to run the model estimation.

### Likelihood ratio test and temporal instability test

Prior to presenting the model results for both unrestrained and restrained driver-injury severity for each period, the paper conducted a series of tests to determine whether the parameter estimates of the restrained driver model are statistically and significantly different from the unrestrained model in each period considered in this study, and to test whether the parameter estimates in restrained and unrestrained driver models are temporally stable or not. To accomplish this, the likelihood ratio test is commonly used ^84, 88^. This test is conducted to either accept or reject the following hypothesis:

H0_1_: In each period, the impacts of parameter estimates are the same between restrained driver-injury and unrestrained driver-injury in speeding-related single-vehicle crashes.

H0_2_: The impacts of parameter estimates for restrained driver-injury or unrestrained driver-injury in speeding-related single-vehicle crashes are temporally stable from one period to the next.

This study used pairwise comparison instead of a global test across all data because it provides direct insight into variability^84^. Suppose $A$ and $B$ are two distinct models using two distinct sub-datasets $A$ and $B$, respectively. According to the previous crash-injury studies^34, 38, 80, 84, 94^, the Chi-square test to compare between two model can be computed as follow:

5 $${\chi }^{2}=-2\left[\mathrm{LL}\left({\upbeta }_{BA}\right)-\mathrm{LL}\left({\upbeta }_{A}\right)\right],$$

where ${\chi }^{2}$ is a Chi-square, $\mathrm{LL}\left({\upbeta }_{BA}\right)$ is a log-likelihood at convergence of the model that used the converged and statistically significant parameters estimated from the B model to analyze dataset of A^84, 88^, and $\mathrm{LL}\left({\upbeta }_{A}\right)$ is a log-likelihood at convergence of the model using the same A subgroup of data, with the same variables as is the case for $\mathrm{LL}\left({\upbeta }_{BA}\right)$ but their parameters (regardless of their statistical significance) are no longer restricted to the converged parameters of subgroup B^84, 88^.

To establish the significance level or confidence level, the resulting ${\chi }^{2}$ distributed with a degree of freedom (equal to the number of significant variables that were used to find $\mathrm{LL}\left({\upbeta }_{BA}\right)$) is used^29, 34^. Table 3 displays the transferability test results between the models for restrained and unrestrained drivers across each time period. As evident from Table 3, all six paired tests demonstrate a relatively high confidence level (over 99%) to refute the initial null hypothesis, stating that the impact of parameter estimates is consistent between restrained and unrestrained driver models for each period. Additionally, Table 4 presents the outcomes of temporal instability tests for every period pair of restrained and unrestrained driver models. Among the 12 tests, only one (A = 2012–2013 restrained driver model, B = 2016–2017 restrained driver model) displays a comparatively low confidence level. Nevertheless, the reversed χ^2^ value of this test (B = 2012–2013 restrained driver model, A = 2016–2017 restrained driver model) successfully rejects the null hypothesis with a high confidence level (over 99%), confirming temporal instability. All other paired tests exhibit confidence levels above 99% to dismiss the null hypothesis, suggesting that the impacts of parameter estimates for restrained or unrestrained driver injuries in speeding-related single-vehicle accidents are not temporally stable throughout the examined period. Consequently, it is essential to divide the entire dataset based on the drivers' seatbelt usage status and distinct timeframes (i.e., 2012–2013, 2014–2015, and 2016–2017) to investigate the factors influencing injury severity in speeding-related single-vehicle crashes.

**Table 3:** Transferability test results between restrained and unrestrained-driver injury severity models.

A =	Restrained driver model	Unrestrained driver model
B =	Unrestrained driver model	Restrained driver model
2012–2013	29.52 (10) [99.89%]	48.61 (9) [99.99%]
2014–2015	55.76 (12) [99.99%]	61.94 (9) [99.99%]
2016–2017	102 (12) [99.99%]	34.45 (14) [99.82%]

**Table 4:** Temporal stability test results of restrained and unrestrained-driver injury severity models.

A/B	Restraint driver model			Unrestraint driver model		
	2012–2013	2014–2015	2016–2017	2012–2013	2014–2015	2016–2017
2012–2013	–	30.48 (9) [99.99%]	17.16 (14) [75.25%]	–	39.53 (12) [99.99%]	67.83 (12) [99.99%]
2014–2015	53.08 (9) [99.99%]	–	41.57 (14) [99.98%]	48.19 (10) [99.99%]	–	101.41 (12) [99.99%]
2016–2017	73.91 (9) [99.99%]	69.69 (9) [99.99%]	–	51.59 (10) [99.99%]	43.89 (12) [99.99%]	–

## Results and discussions

In this study, four distinct parameter density functions are pre-specified, as indicated in Eq. (2), which encompass normal, triangular, uniform, and lognormal distributions. Table 5 illustrates the comparison results of the estimated models with varying distribution assumptions. This table reveals that five individual models generated one random parameter with four random distributions. Furthermore, it demonstrates that the log-likelihood function and AIC at convergence for the mixed logit model with a normal distribution are marginally superior to those employing triangular, uniform, and lognormal distributions. In comparison to other distributions, the normal distribution excels at capturing the central tendency and variations of random variables concerning driver injury severity probability ^25, 47^.

**Table 5:** Comparison results of models with different distributions.

Model	Random parameters	Distribution types			
		Normal	Triangular	Uniform	Lognormal
2012–2013 Restraint driver model	Male [MI]	LL(β) = −522.50 AIC = 1117	LL(β) = −522.53 AIC = 1117.06	LL(β) = −522.60 AIC = 1117.2	(Fail to converge)
2014–2015 Restraint driver model	Four-lane [MI]	LL(β) = −661.48 AIC = 1394.96	LL(β) = −661.50 AIC = 1395	LL(β) = −661.49 AIC = 1394.98	LL(β) = −662.28 AIC = 1396.56
2016–2017 Restraint driver model	Four-lane [MI]	LL(β) = −563.49 AIC = 1200.98	LL(β) = −563.50 AIC = 1201	LL(β) = −563.59 AIC = 1201.18	(Fail to converge)
2014–2015 Unrestraint driver model	Four-lane [MI]	LL(β) = −1223.61 AIC = 2521.2	LL(β) = −1223.65 AIC = 2521.3	LL(β) = −1223.67 AIC = 2521.4	LL(β) = −1225.93 AIC = 2525.9
2016–2017 Unrestraint driver model	Asphalt [MI]	LL(β) = −1011.36 AIC = 2094.72	LL(β) = −1011.40 AIC = 2094.8	LL(β) = −1011.41 AIC = 2094.82	LL(β) = −1011.51 AIC = 2095.02

The model results for restrained and unrestrained driver-injury models by periods are presented in Tables 6 and 7, respectively. Moreover, the summary of marginal effects of significant factors for restrained and unrestrained driver-injury models is shown in Tables 8 and 9, respectively. Out of the six models, only the 2012–2013 unrestrained driver model did not produce statistically significant random parameters, leading the model to revert to the standard multinomial logit model. The other five models produced random parameters with heterogeneity in means, while only the 2014–2015 unrestrained driver model displayed heterogeneity in variance. All models exhibited relatively high McFadden Pseudo R^2^ values (>0.3), which are considered acceptable compared to existing research ^84, 95^. The following subsections provide a discussion of the results based on the average marginal effect and the coefficient (in the case of random parameters). All parameters presented in the tables have a significance level of 0.1 or lower (indicated by the t-Stat), as this level is considered relatively important to the outcome of injury-severity probabilities^32^.

**Table 6:** Mixed logit with heterogeneity in mean modeling for restrained driver-injury severity model in speeding-related single vehicle crash (parameters defined for [MI] = Minor injury; [SI] = Severe injury; [FI] = Fatal injury).

Variable	2012–2013		2014–2015		2016–2017	
	Coefficient	t-Stat	Coefficient	t-Stat	Coefficient	t-Stat
Constant [SI]	−1.320	−1.94	−0.853	−1.80	−3.834	−4.03
Constant [FI]	−1.348	−1.96	−1.683	−1.60	−3.351	−3.70
*Male [MI]*	0.233	0.38				
*SD "Male"*	2.082	1.71				
Alcohol [FI]			1.869	2.83		
Flush median [FI]	1.567	1.82	1.391	2.99		
Flush median [MI]					−1.674	−1.78
Barrier median [SI]			0.902	1.99		
Barrier median [MI]					−1.487	−2.90
*Four-lane [MI]*			1.304	1.77	1.977	2.25
*SD "Four-lane"*			1.968	1.68	2.993	2.73
Asphalt [MI]					−1.927	−2.66
Slope [MI]	−1.798	−2.2				
Slope [SI]					−1.036	−2.03
Intersection [FI]			−2.314	−2.21		
Van [MI]					−1.630	−1.85
RORS [MI]	−2.289	−3.74	−1.480	−2.82	−1.634	−3.91
RORSG [SI]	0.779	2.18				
RORSG [FI]					−1.204	−3.52
RORC [MI]	−1.464	−2.22			−1.546	−2.80
RORC [FI]			1.141	3.10		
RORCG [FI]	−0.983	−2.17			−0.974	−2.58
MTI [FI]					−1.385	−3.03
Unlit road [SI]					0.683	1.77
Lit road [MI]			−0.431	−1.73		
Weekend [FI]	0.586	2.03				
**Heterogeneity in means**						
Male : Slope	1.482	1.64				
Four-lane : Depressed median			−1.201	−2.53		
Four-lane : Old					−1.645	−2.10
Four-lane : Flush median					2.729	2.04
**Model statistic**						
Number of observations	773		999		842	
LL(0)	−849.227		−1097.514		−925.032	
LL(β)	−522.502		−661.488		−563.492	
McFadden Pseudo R^2^	0.385		0.397		0.391

**Table 7:** Mixed logit with heterogeneity in mean modeling for unrestrained driver-injury severity model in speeding-related single vehicle crash (parameters defined for [MI] = Minor injury; [SI] = Severe injury; [FI] = Fatal injury).

Variable	2012–2013		2014–2015		2016–2017	
	Coefficient	t-Stat	Coefficient	t-Stat	Coefficient	t-Stat
Constant [SI]	−1.177	−2.12	0.038	2.07	−1.273	−2.26
Constant [FI]	−1.049	−1.8	−0.552	−2.22	−0.762	−2.45
Young [MI]					1.049	1.74
Old [FI]	0.498	2.01				
Alcohol [MI]	−2.278	−2.04				
Raised Median [FI]	0.597	2.86				
Raised median [MI]			−0.314	−1.81		
Raised mediant [SI]					0.623	2.40
Depressed median [FI]			0.521	2.94		
Barrier Median [SI]	0.843	2.1				
*Four-lane [MI]*			1.219	2.13		
*SD "Four-lane"*			1.461	2.00		
*Asphalt [MI]*			0.432	1.68	1.940	2.41
*SD "Asphalt"*					3.727	2.68
Slope [MI]	−0.439	−1.86				
Slope [SI]			0.554	2.05		
Intersection [FI]					−1.213	−2.10
U-turn [FI]			−1.820	−2.59		
U-turn [MI]					1.600	2.50
Passenger car [SI]					0.553	2.19
RORS [MI]	−0.416	−1.84				
RORS [FI]			0.917	4.18		
RORS [SI]					−0.978	−3.05
RORSG [FI]	−0.774	−3.18				
RORSG [MI]			0.821	3.64	1.494	3.72
RORC [SI]					−1.007	−2.37
RORCG [MI]					0.971	1.83
MTI [FI]	−0.995	−3.76				
MTI [MI]			0.965	3.48		
Raining [MI]			−0.962	−1.75	−1.629	−1.85
Morning peak hour [SI]	−0.981	−2.05				
Evening peak hour [MI]	0.455	1.92				
**Heterogeneity in means**						
Four-lane : Male			−0.762	−1.78		
Asphalt : Young					−1.283	−1.77
**Heterogeneity in variances**						
Four-lane : Old			0.781	1.71		
**Model statistic**						
Number of observations	1228		1608		1387	
LL(0)	−1349.096		−1766.569		−1523.775	
LL(β)	−914.609		−1223.616		−1011.362	
McFadden Pseudo R^2^	0.322		0.307		0.336

**Table 8:** Summary of marginal effect for significant factors in the restrained driver-injury severity models.

Variable	2012–2013			2014–2015			2016–2017		
	Minor	Severe	Fatal	Minor	Severe	Fatal	Minor	Severe	Fatal
Male	−0.0620	0.0396	0.0224						
Alcohol				−0.0039	−0.0009	0.0048			
Flush median	−0.0034	−0.0012	0.0047	−0.0066	−0.0021	0.0087	−0.0070	0.0037	0.0033
Barrier median				−0.0050	0.0059	−0.0009	−0.0168	0.0093	0.0075
Four-lane				−0.0151	0.0110	0.0042	0.0070	0.0000	−0.0070
Asphalt							−0.1998	0.1079	0.0918
Slope	−0.0236	0.0151	0.0085				0.0039	−0.0062	0.0023
Intersection				0.0016	0.0007	−0.0023			
Van							−0.0049	0.0030	0.0020
RORS	−0.0408	0.0183	0.0224	−0.0267	0.0166	0.0101	−0.0334	0.0129	0.0205
RORSG	−0.0182	0.0243	−0.0061				0.0134	0.0102	−0.0236
RORC	−0.0125	0.0059	0.0065	−0.0087	−0.0026	0.0113	−0.0134	0.0038	0.0096
RORCG	0.0062	0.0036	−0.0098				0.0123	0.0046	−0.0169
MTI							0.0069	0.0057	−0.0126
Unlit road							−0.0053	0.0077	−0.0024
Lit road				−0.0224	0.0134	0.0090			
Weekend	−0.0100	−0.0054	0.0154

**Table 9:** Summary of marginal effect for significant factors in the unrestrained driver-injury severity models.

Variable	2012–2013			2014–2015			2016–2017		
	Minor	Severe	Fatal	Minor	Severe	Fatal	Minor	Severe	Fatal
Young							0.0188	−0.0100	−0.0087
Old	−0.0077	−0.0017	0.0095						
Alcohol	−0.0015	0.0007	0.0008						
Raised median	−0.0189	−0.0044	0.0232	−0.0138	0.0075	0.0064	−0.0086	0.0153	−0.0067
Depressed median				−0.0157	−0.0055	0.0212			
Barrier median	−0.0041	0.0047	−0.0006						
Four-lane				−0.0080	0.0062	0.0018			
Asphalt				0.0619	−0.0313	−0.0306	−0.0199	0.0107	0.0091
Slope	−0.0101	0.0047	0.0055	−0.0057	0.0072	−0.0015			
Intersection							0.0012	0.0019	−0.0031
U-turn				0.0020	0.0012	−0.0032	0.0083	−0.0051	−0.0032
Passenger car							−0.0100	0.0198	−0.0098
RORS	−0.0107	0.0045	0.0062	−0.0169	−0.0069	0.0238	0.0031	−0.0080	0.0049
RORSG	0.0178	0.0040	−0.0218	0.0381	−0.0198	−0.0183	0.0379	−0.0207	−0.0172
RORC							0.0015	−0.0044	0.0029
RORCG							0.0184	−0.0098	−0.0086
MTI	0.0180	0.0035	−0.0214	0.0307	−0.0172	−0.0135			
Raining				−0.0350	0.0177	0.0173	−0.0147	−0.0156	0.0303
Morning peak hour	0.0031	−0.0037	0.0006						
Evening peak hour	0.0075	−0.0041	−0.0034						

### Driver-related variables

It is well established in the literature that age serves as a proxy for the physiological and behavioral characteristics of drivers that are likely to statistically influence crash severity ^35, 83^. In this study, two driver age groups were utilized: young drivers (aged under 26 years old) and old drivers (aged over 49 years old). As seen in Table 7, the variable reflecting young drivers was found to be statistically significant in the 2016–2017 unrestrained driver model, with the marginal effect showing a higher likelihood of minor injury (0.0188) and lower likelihood of severe and fatal injuries (−0.0100 and −0.0087, respectively). Conversely, the variable representing old drivers was found to be a significant factor in the 2012–2013 unrestrained driver model, with the marginal effect indicating a higher likelihood of fatal injury (0.0095) and lower likelihood of other severity levels. This finding is intuitive since older drivers may have weaker physiques (lower injury tolerances) and weaker visual acuity, as well as possibly slower reaction times to avoid crashes compared to younger drivers ^74, 96^.

In terms of indirect effects, the indicator for old drivers was found to decrease the mean of the variable reflecting crashes on four-lane roadways (random parameter) in the 2016–2017 restrained driver model. In simpler terms, even while wearing seatbelts, old drivers involved in speeding-related crashes on four-lane roads are less likely to experience minor injuries and more likely to suffer severe and fatal injuries. Similarly, in the 2016–2017 unrestrained driver model, the indicator for young drivers was found to decrease the mean of the variable reflecting crashes on roads with asphalt pavement (random parameter), making minor injuries less likely and severe or fatal injuries more likely. Furthermore, in the 2014–2015 unrestrained driver model, the indicator for old drivers was found to generate significant heterogeneity in variance. This means it increased the variance of the random parameter (i.e., indicator for crashes on four-lane roadways), widening the random parameter's effect distribution or increasing variability. Regarding temporal instability, the old driver variable was significant only in the 2012–2013 period (having a higher risk of fatal injury) and insignificant in later periods. This may indicate a slight improvement for unrestrained old drivers resulting from advancements in other vehicle safety features over time, such as improvements in braking systems, airbags, or stability control^29^.

Regarding the gender of drivers, in the 2012–2013 restrained driver model, the variable reflecting male drivers resulted in a significant random parameter (defined for minor injury) with a mean = 0.233 and standard deviation = 2.082. This distribution indicates that 54.46% of the seatbelt-restrained male drivers were more likely to experience minor injuries, whereas 45.54% of them were more likely to experience severe or fatal injuries in speeding-related crashes. However, the average marginal effect (Table 8) shows that restrained male drivers are more likely to sustain severe or fatal injuries in speeding-related crashes compared to their female counterparts. This suggests that although the proportion of severe or fatal injuries is slightly less than minor injuries, the probability magnitude of severe and fatal injuries in each crash for the 45.54% of male drivers is comparatively higher than that for the 54.46% of male drivers. The random parameter reflecting male drivers may be due to variations in physiological characteristics and safety and risk awareness ^74, 75^. Such heterogeneous effects of gender-related variables on crash severity outcomes have also been reported in previous studies^91, 97^. In terms of temporal instability, the male indicator was found significant only in the 2012–2013 restrained model and became insignificant in later periods. This is likely due to the improvement of other safety features in vehicles over time^29^.

Lastly, the variable reflecting drivers under the influence of alcohol was found to be statistically significant in the 2012–2013 unrestrained driver model and the 2014–2015 restrained driver model, consistently indicating a higher likelihood of fatal injury in speeding-related crashes. Previous studies have also reported similar findings^4, 37, 98^. As for temporal instability, the reason this variable was not found statistically significant in later periods may be due to the strengthening of law enforcement on drunk driving in Thailand through the Road Traffic Act (2014), which may have influenced driving behavior and drivers' awareness of police checkpoints due to stricter penalties^4, 32^.

### Roadway-related attributes

In this study, crashes on different road median types were also considered and found to affect the resulting driver-injury severity. In only the restrained driver models (Table 6), variable reflecting speeding-related crashes on roadway with flush median was found to be statistically significant in all three periods model (2012–2013, 2014–2015, and 2016–2017), with a relatively stable average marginal effect having a higher likelihood of fatal injury (as seen in Table 8). A potential reason for the flush median's prevalence in Thailand could be its frequent use in rural areas, where speed limits are typically higher and traffic volume is considerably lower compared to urban regions. The significance of this variable in the restrained driver model might be attributed to the likelihood of drivers traversing rural areas compensating for the safety advantages gained from wearing seat belts by adopting a more negligent and aggressive driving behaviors (offsetting behavior)^65, 99^. On the other hand, the variable representing crashes on roadways with raised medians was found to be a significant factor in all period models for unrestrained drivers only. According to the average marginal effect in Table 9, crashes on roadways with raised medians increased the likelihood of fatal injury in the 2012–2013 model, increased the likelihood of both severe and fatal injuries in the 2014–2015 model, and increased the likelihood of severe injury only in the 2016–2017 model. The variable representing crashes on roadways with depressed medians was significant only in the 2014–2015 unrestrained model, with the average marginal effect increasing the likelihood of fatal injury (0.0212). The variable representing crashes on roadways with barrier medians was found to be a significant factor in the 2012–2013 unrestrained driver, 2014–2015 restrained driver, and 2016–2017 restrained driver models, with stable average marginal effects across all models increasing the likelihood of severe injury (0.0047, 0.0059, and 0.0093, respectively). These findings are particularly important because they show that the effect of road medians significantly influences driver-injury severity in speeding-related single-vehicle crashes. Raised medians and barrier medians are built in urban areas or on roads approaching urban areas (or municipal areas). Some of the benefits of both median types include reducing crash frequency (by separating opposing streams of traffic and restricting turning movements) and reducing vehicle speeds on the roadway ^100, 101^. However, if drivers encounter a crash on these roads due to speeding, they face an increased possibility of being involved in a severe or even fatal crash. The significance of the barrier median roadway effect only in the restrained driver model is mainly due to the widespread use of barrier medians in rural areas with many curved or mountainous roads in Thailand, in order to prevent head-on collisions. As a result, the significance of this variable in the model for restrained drivers may be due to drivers driving on rural roads who negate the safety advantages of wearing seat belts by exhibiting more reckless and aggressive driving behavior (similar to the effect of flush median roadways). In the past literature, Al-Bdairi and Hernandez ^101^ found that over 20% of run-off-road crashes on roadways with raised medians are more likely to result in severe crashes. However, some studies have found that raised medians reduce the rate of severe crashes by over 30% ^102, 103^. The randomness of this finding suggests that a cohort of crashes on roads with raised medians may lead to a higher risk of death and serious injuries due to a reduction in the effectiveness of vehicle safety features and the benefits of raised medians because of speeding ^4^.

Interestingly, the effect of the number of lanes showed significant variability across crash observations in both unrestrained and restrained driver-injury severity models. In the 2014–2015 restrained driver models, the variable representing crashes on four-lane roads was found to generate a significant random parameter for minor injuries. With a mean of 1.304 and a standard deviation of 1.968, the results indicated that 74.62% of crashes on four-lane roads had a higher likelihood of minor injuries, while 25.38% had a higher likelihood of severe or fatal injuries. Similarly, in the 2016–2017 restrained driver model, the crash indicator on four-lane roads also yielded a significant random parameter for minor injuries, with a mean of 1.977 and a standard deviation of 2.993, suggesting that 74.55% of crashes on four-lane roads had a higher likelihood of minor injuries and 25.45% had a higher likelihood of severe or fatal injuries. In the 2014–2015 unrestrained driver model, the variable representing crashes on four-lane roads resulted in a random parameter for minor injuries, with a mean of 1.219 and a standard deviation of 1.461, indicating that 79.8% of crashes on four-lane roads had a higher likelihood of minor injuries and 20.2% had a higher likelihood of severe or fatal injuries. The emergence of this variable as a random parameter is logical, as most documented speeding-related accidents occurred on four-lane highways (as shown in Table 2), which can display considerable variation in factors such as crash types, crash mechanisms, and vehicle types/conditions. The subset of four-lane roadway crashes with an increased probability of serious or fatal injuries could be influenced by unobservable factors, such as a group of highly aggressive drivers, older vehicle models, or extreme crash scenarios (e.g., rollovers), which remain undetected by the analyst.

The variable representing crashes on asphalt pavement was found to be significant in the 2014–2015 unrestrained driver model, the 2016–2017 restrained driver model, and the 2016–2017 unrestrained driver model, exhibiting temporal instability across the periods examined. Specifically, it was associated with a higher likelihood of minor injuries in 2014–2015, while increasing the likelihood of severe and fatal injuries for both restrained and unrestrained drivers in the 2016–2017 models. It is important to note that in the 2016–2017 unrestrained driver model, this variable emerged as a significant random parameter (defined for minor injury), with a mean of 1.940 and a standard deviation of 3.727. This distribution suggests that 69.86% of crashes on roads with asphalt pavement had a likelihood of minor injury, while 30.14% had a higher likelihood of severe or fatal injuries. Although the effect transitioned to a higher probability of severe or fatal injuries in the later period, the underlying cause remains uncertain. Further investigation is necessary to determine whether this pattern persists or changes in years following 2017.

In the restrained driver models, variables representing crashes on sloped roads emerged as significant factors in both 2012–2013 and 2016–2017, with the average marginal effect increasing the likelihood of severe injury in 2012–2013 and fatal injury in 2016–2017 (as seen in Table 8). Similarly, this variable was also significant in the 2012–2013 and 2014–2015 unrestrained driver models, with the average marginal effect increasing the likelihood of fatal injury in 2012–2013 and severe injury in 2014–2015.

The variable representing crashes at intersection areas was found to be statistically significant in the 2014–2015 restrained driver and 2016–2017 unrestrained driver models, with a consistent average marginal effect increasing the likelihood of severe and minor injuries. Lastly, the variable reflecting crashes at U-turn areas was significant only in the 2014–2015 and 2016–2017 unrestrained driver models, with the average marginal effect increasing the likelihood of both severe and minor injuries.

### Vehicle types-related factors

Regarding vehicle types, the variable for van drivers was significant only in the 2016–2017 restrained driver model, leading to a higher likelihood of severe and fatal injuries (Table 8). Conversely, the variable for passenger cars was significant solely in the 2016–2017 unrestrained driver model, resulting in a higher likelihood of severe injuries in speeding-related crashes.

### Crash-related characteristics

Regarding variables from the crash characteristics, both restrained and unrestrained driver-injury severity models exhibited similar results with only differences in the value of the marginal effects. In every time period for both restrained and unrestrained driver models, the variable for crashes involving vehicles running off a straight roadway (without a roadside guardrail) was found to be statistically significant, consistently increasing the likelihood of severe or fatal injuries (Tables 8, 9). However, for all periods of both restrained and unrestrained driver models (except the 2014–2015 restrained driver model), crashes involving vehicles running off a straight roadway and subsequently hitting a roadside guardrail were found to significantly reduce the likelihood of fatal and severe injuries in speeding-related crashes (Tables 8, 9). Similarly, the variable for crashes involving vehicles running off a curved roadway (without a roadside guardrail) was found to be statistically significant in all periods of restrained driver models and the 2016–2017 unrestrained driver model, consistently increasing the likelihood of fatal injuries. In contrast, crashes involving vehicles running off a curved roadway and hitting a roadside guardrail were found to increase the likelihood of minor injuries while decreasing the risk of fatal injuries for 2012–2013 restrained drivers, 2016–2017 restrained drivers, and 2016–2017 unrestrained driver models. This result is in line with previous studies and makes intuitive sense ^53, 64, 104^, highlighting the crucial safety advantages of roadside guardrail protection in mitigating hazardous crash mechanisms such as rollover or overturn crashes, preventing vehicles from going off the road, absorbing crash energy, and reducing the consequences of driving errors on forgiving roads ^105^. Lastly, crashes that involve a vehicle running over a traffic island were found to be significant in the 2016–2017 restrained driver and 2012–2013 and 2014–2015 unrestrained driver models. The average marginal effect of this variable indicates a higher likelihood of minor or severe injuries.

### Environmental and temporal-related factors

In terms of weather conditions, the variable representing crashes occurring in rainy conditions was identified as a significant factor exclusively in the 2014–2015 and 2016–2017 unrestrained driver models. The consistent average marginal effect of this variable increased the probability of fatal injuries in crashes. Past research has also demonstrated a strong association between rainy weather and heightened severity in single-vehicle accidents^4, 49^. Similarly, crashes on unlit road and lit road were found as a significant factor in 2016–2017 and 2014–2015 restrained driver model, respectively. Both types of these crashes have a higher likelihood of severe injury (as seen in Table 8). Previous studies also reported that crashes at nighttime have a higher probability of severe injury compared to daytime crashes ^85, 106, 107^. A potential explanation for the insignificance of this variable in unrestrained driver models could be the tendency of these drivers to adopt more careless and aggressive driving habits as a result of the perceived safety boost they gain from wearing seat belts^65^. Variable representing crashes at weekend was significant in only 2012–2013 restrained driver model, with the effect increasing the likelihood of fatal injury. Lastly, crashes during morning and evening peak hour were significant in only 2012–2013 unrestrained driver models, with the effect increasing the likelihood of minor injury (Table 9).

### Insights from out-of-sample prediction simulation

In the crash-injury severity research, the concept of the out-of-sample prediction is used to compare the outcomes’ predicted probabilities of two or more crash-injury models^84^. The test uses the full parameter estimates of an A crash model (with predefined probabilities based on an A data) to predict the injury outcome of a B data (in this case study, A and B could represent restrained and unrestrained crash model/data or period A/B, respectively). For example, Islam et al.^95^ applied the simulation and found that, with the same crash’s associated characteristics, the crashes in 2017 would produce 3.8% less number of minor injuries and 0.5% less rate of severe injuries, compared to crashes in 2012 (the study identified the improvement in vehicle safety feature over time as the possible cause of these changes). With the use of the simulation, Alogaili and Mannering ^78^ found that pedestrian-vehicle crashes in the daytime would cause as much as 16.45% less severe injuries compared to the crashes at nighttime, given both crash times having the same other associated factors. Additionally, the application of this simulation has also been adopted by numerous recent crash severity studies to gain a better overview understanding of how two or more crash conditions are different in influencing injury severities^38, 40, 80, 90^. Likewise, this study also adopted this simulation for predictive comparison between restrained and unrestrained driver-injury severities and investigating how injury severity distribution changed over time. The result of this simulation will seek an answer to these fundamental questions: (1) “*what would the unrestrained driver-injury severity distribution have been if the restrained driver models’ parameter estimates were utilized to predict them?*” and (2) “*What would have been the injury severity distribution for the later-period crashes if previous-period estimated model parameter were used to forecast them?”*. Theoretically, since a mixed logit model with means and variances heterogeneity was used in this study, it is recommended to fully account for both means and variances of the random parameters in the simulation to eliminate inaccurate prediction^84^. The out-of-sample prediction simulation can be computed by^84^,

$${P}_{i}\left(n\right)\approx \frac{1}{K}\sum_{k=1}^{K}\frac{{e}^{\left\{\left[{{\varvec{\beta}}}_{{\varvec{n}}}+{\Theta }_{in}{{\varvec{M}}}_{in}+{\sigma }_{in}EXP\left({{\varvec{\omega}}}_{in}{{\varvec{S}}{\varvec{D}}}_{in}\right){\nu }_{in,k}\right]{X}_{in}\right\}}}{{\sum }_{n=1}^{N}{e}^{\left\{\left[{{\varvec{\beta}}}_{{\varvec{n}}}+{\Theta }_{in}{{\varvec{M}}}_{in}+{\sigma }_{in}EXP\left({{\varvec{\omega}}}_{in}{{\varvec{S}}{\varvec{D}}}_{in}\right){\nu }_{in,k}\right]{X}_{in}\right\}}}$$

where all terms are previously defined and $K$ is the total number of Halton draws for individual observation (as indicated in the earlier section, 1000 Halton draws were used to obtain stable parameters).

Table 10 displays the difference between out-of-sample predictions (i.e., using restrained driver-injury model parameters to predict injury severity outcomes with data from unrestrained drivers) and within-sample predictions (i.e., using unrestrained driver-injury model parameters to predict injury severity outcomes with data from unrestrained drivers) for unrestrained driver injury severity, using the restrained driver model as the baseline. Specifically, when using the restrained driver model to predict unrestrained driver injury severity, minor injuries will be overestimated by 0.0815, 0.0933, and 0.0858 during the periods of 2012–2013, 2014–2015, and 2016–2017, respectively. In contrast, severe injuries will be underestimated by −0.0041 in 2012–2013, overestimated by 0.0093 in 2014–2015, and underestimated by −0.0065 in 2016–2017. Most notably, the distribution of fatal injuries will be significantly underestimated during all periods by −0.0774, −0.1026, and −0.0793, respectively. In more straightforward terms, if the contributing factors were identical for each crash involving drivers not wearing seatbelts, the parameters estimated for restrained drivers would predict a considerably lower number of severe and fatal crashes than what was actually observed. These simulation results clearly demonstrate that wearing seatbelts provides a substantial safety advantage, which could greatly reduce the fatality rate resulting from speeding-related single-vehicle run-off-road crashes.

**Table 10:** Means of probability differences in predicting the injury-severity between different restrained and unrestrained drivers.

Base model	Injury	Forecast model: Unrestrained driver-injury model		
		2012–2013	2012–2014	2012–2015
Restrained driver-injury model	Minor	0.0815	0.0933	0.0858
	Severe	−0.0041	0.0093	−0.0065
	Fatal	−0.0774	−0.1026	−0.0793

Regarding the overall impact of temporal instability, Table 11 shows the differences between out-of-sample and within-sample prediction probabilities for later periods, using earlier-period models as the baseline. For restrained drivers, using the 2012–2013 model to predict 2014–2015 outcomes would result in overestimating fatal injuries by 0.002 while underestimating minor and severe injuries by −0.001 each. However, when applying the 2012–2013 model to forecast 2016–2017, minor injuries would be overestimated by 0.0297, while severe and fatal injuries would be underestimated by −0.0083 and −0.0214, respectively. When using the 2014–2015 model to predict 2016–2017, minor and severe injuries would be overestimated by 0.0297 and 0.0012, respectively, and fatal injuries would be underestimated by −0.0309. For unrestrained drivers, using the 2012–2013 model to predict 2014–2015 and 2016–2017 would consistently overestimate minor and severe injuries, while underestimating fatal injuries (as shown in Table 11). In contrast, using the 2014–2015 model to predict 2016–2017 would underestimate minor injuries by −0.005 and overestimate severe and fatal injuries by 0.0007 and 0.0043, respectively. Overall, the out-of-sample prediction findings highlight the considerable combined impact of temporal instability and the non-transferability of restrained and unrestrained driver injury severities across the periods studied.

**Table 11:** Means of probability differences in predicting the injury-severity between different period for restrained and unrestrained drivers.

Base years	Injury	Forecast years			
		Restraint model		Unrestraint model	
		**2014–2015**	2016–2017	2014–2015	2016–2017
2012–2013	Minor	−0.0010	0.0297	0.0168	0.0043
	Severe	−0.0010	−0.0083	0.0044	0.0036
	Fatal	0.0020	−0.0214	−0.0212	−0.0079
2014–2015	Minor	–	0.0297	–	−0.0050
	Severe	–	0.0012	–	0.0007
	Fatal	–	−0.0309	–	0.0043

## Summary and conclusions

Using a mixed logit model with heterogeneity in means and variances, this paper examines and compares the differences in injury severity between unrestrained and restrained drivers in speed-related single-vehicle crashes, accounting for temporal instability. The data used in the study was obtained from the Department of Highways and covers a period of six years, divided into three time periods: 2012–2013, 2014–2015, and 2016–2017. The study considers three levels of injury severity: minor injury, severe injury, and fatal injury. In addition, various risk factors such as driver characteristics, road conditions, vehicle factors, crash characteristics, and environmental and temporal factors were taken into account in the analysis.

Two series of likelihood ratio tests showed that the estimated parameters between the unrestrained and restrained driver-injury models were non-transferable and exhibited temporal instability across the studies period. For restrained drivers, the risk of fatal or severe crashes was positively associated with factors, such as male drivers, under influence of alcohol, flush/barrier median roadway, slope roadway, van, running off roadway without roadside guardrail, and nighttime on unlit/lit road. For unrestrained drivers, the likelihood of fatal or severe injury increases for old drivers’ crashes, under influence of alcohol, raised/depressed median roadways, four-lane roadway, passenger car, running off roadway without roadside guardrail, and crash under rainy condition. Lastly, the out-of-sample prediction simulation findings are particularly important. It shows the upper limit of safety benefits that can be achieved by just using a vehicle’s seatbelt system. Alternatively, this finding illustrated a potential reduction in the rate of severe and fatal injuries by just replicating restrained driver conditions.

There are several key insights from the study. Firstly, old drivers linked to fatal crashes (in the model for unrestrained drivers) were determined to be significant only in earlier periods. This might imply that, for drivers not using seat belts, advancements in other vehicle safety features like improved braking systems, airbags, or stability control systems could also contribute to temporal instability. Hence, ongoing efforts to encourage individuals to use newer vehicles equipped with high-quality safety features, besides seat belts, could potentially help prevent drivers from involving in severe and fatal speeding-related accidents over time. Promote the adoption of newer vehicles equipped with safety features such as adaptive cruise control, lane-keeping assist, and automatic emergency braking, which can help prevent speeding-related crashes. Secondly, it was observed that unrestrained drivers under the influence of alcohol were significantly associated with fatal crashes only in earlier periods (but not in 2014–2015 and 2016–2017). The temporal instability may be attributed to the increased rigor of law enforcement on drunk driving in Thailand, as a result of the Road Traffic Act (2014). Therefore, consistent reviewing and improving this legislation may have impacted driving behavior and drivers' awareness of police checkpoints due to the imposition of stricter penalties. Thirdly, drivers who wear seat belts and are involved in speeding-related accidents on roads with flush and barrier consistently exhibit a higher likelihood of experiencing severe and fatal injuries over the observed periods (however, these factors were not deemed significant in models for unrestrained drivers). A possible reason for this could be that drivers may offset the benefits of using seat belts by adopting more aggressive driving behaviors. This suggests a necessity for creating informational initiatives on seat belt usage that emphasize not only the significant protective advantages of wearing seat belts, but also the risks posed to other motorists by aggressive driving. Gaining a deeper comprehension of the neuropsychological and cognitive processes that drive aggressive driving behavior, as well as how individuals perceive their obligations to others in relation to their own safety, can be valuable in developing strategies to curb aggressive driving^65^. Fourthly, it appears that speeding-related accidents on sloped roads consistently raise the probability of fatal injuries for both restrained and unrestrained drivers. As a result, possible solutions include enforcing stricter speed limits on sloped roads, integrating features like skid-resistant surfaces, improved drainage, and enhanced visibility, as well as ensuring that clear and conspicuous road signs—such as warnings for steep inclines, declines, and sharp turns—are in place to assist drivers in safely navigating sloped roads. Fifthly, the presence of roadside guardrails consistently reduces the risk of severe and fatal injuries for both restrained and unrestrained drivers over the considered periods. Guardrails serve as a protective barrier that absorbs and redistributes the impact forces from a collision, which can prevent vehicles from veering off the road, rolling over, or colliding with roadside hazards such as trees or poles. Thus, a potential countermeasure would involve increasing the installation of roadside guardrails to compensate for drivers' errors and aggressive driving behaviors, enhancing overall road safety and potentially saving lives. Lastly, a combination of speeding-related crashes during rainy conditions and unrestrained drivers has been associated with a higher probability of fatal injuries in the most recent two periods. Therefore, it is necessary to raise public awareness by conducting campaigns to emphasize the risks of speeding and not wearing seat belts during rainy conditions and educating drivers about safe driving practices in adverse weather.

### Limitation of the study

This study possesses certain limitations. It is unable to provide the estimated vehicle speed prior to crash, as this information could not be collected by the police. The significance of this limitation lies in the fact that a slight excess of 1 mph over the speed limit, compared to a more substantial 20 mph excess, can drastically influence the severity of injuries sustained. Consequently, it is recommended that future research endeavours differentiate cases based on distinct speeding categories, which could potentially reveal insightful information regarding the relationship between contributing factors and injury severity.

## Data Availability

The datasets generated during the current study will be made available from the corresponding author on reasonable request.
